# Aneurysm Shape and Sac Shrinkage After Total Arch Replacement With Frozen Elephant Trunk for True Aortic Arch Aneurysm

**DOI:** 10.1093/icvts/ivaf200

**Published:** 2025-08-21

**Authors:** Shinji Abe, Yasushige Shingu, Taro Minamida, Nobuyasu Kato, Hiroshi Sugiki, Satoru Wakasa

**Affiliations:** Department of Cardiovascular Surgery, Oji General Hospital, Tomakomai, 053-8506, Japan; Department of Cardiovascular Surgery, Faculty of Medicine and Graduate School of Medicine, Hokkaido University, Sapporo 060-8648, Japan; Department of Cardiovascular Surgery, Faculty of Medicine and Graduate School of Medicine, Hokkaido University, Sapporo 060-8648, Japan; Department of Cardiovascular Surgery, Faculty of Medicine and Graduate School of Medicine, Hokkaido University, Sapporo 060-8648, Japan; Department of Cardiovascular Surgery, Faculty of Medicine and Graduate School of Medicine, Hokkaido University, Sapporo 060-8648, Japan; Department of Cardiovascular Surgery, Faculty of Medicine and Graduate School of Medicine, Hokkaido University, Sapporo 060-8648, Japan

**Keywords:** frozen elephant trunk, true arch aneurysm, sac shrinkage

## Abstract

**Objectives:**

This study aimed to evaluate midterm outcomes and identify predictors of sac shrinkage following total arch replacement with a frozen elephant trunk (TAR-FET) for true aortic arch aneurysms.

**Methods:**

A retrospective analysis was conducted on 28 patients who underwent elective TAR-FET for true arch aneurysms between July 2014 and March 2022. Postoperative sac changes on CT were categorized as shrinkage (≥5 mm reduction), enlargement (≥5 mm increase), or no change (<5 mm change). The sphericity index, a novel morphological parameter, was calculated by dividing the average axial and sagittal sac diameters by sac length.

**Results:**

The median age was 74 years, and 23 were male. No operative deaths or recurrent laryngeal nerve palsy occurred. Among 28 patients, 12 experienced sac shrinkage attributable to the initial TAR-FET. Over a median follow-up of 3.6 years, 12 cases showed shrinkage, 2 enlargement, and 14 no change. Cumulative shrinkage rates at 1, 2, and 3 years were 42%, 47%, and 47%, respectively. Additional thoracic endovascular aortic repair (TEVAR) was required in 5 patients (22% at 3 years). Multivariable analysis showed that shorter preoperative sac length (subdistribution hazard ratio [SHR] 0.96; 95% CI, 0.93-0.99) and a higher sphericity index (per 0.1 increment: SHR 1.38; 95% CI, 1.21-1.57) were independently associated with sac shrinkage.

**Conclusions:**

TAR-FET resulted in sac shrinkage in a substantial proportion of patients. Aneurysms that were shorter and more protruding, as indicated by a higher sphericity index, may be more likely to exhibit postoperative sac shrinkage.

**Clinical Registration Number:**

022-0242; 16 November 2022 (Ethics Committee of Hokkaido University Hospital).

## Graphical abstract

**Figure:**
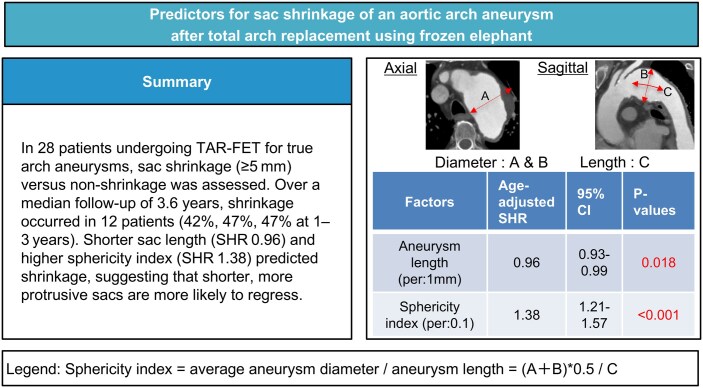

## INTRODUCTION

The frozen elephant trunk (FET) technique, initially reported by Kato et al,^1^ was developed to facilitate the management of thoracic aortic aneurysms and dissections using a stented graft. Its integration into total arch replacement (TAR) has since emerged as a pivotal strategy for complex aortic diseases, simplifying distal anastomosis by enabling proximalization. While TAR with FET (TAR-FET) promotes favourable remodelling in acute type A dissections by inducing false lumen thrombosis,^2^^,^^3^ its role in true aortic arch aneurysms remains less clearly defined. Especially in cases of aortic arch aneurysms extending into the proximal descending aorta, staged or extensive thoracotomy approaches may be necessary and are often technically demanding.^4^ In such cases, TAR-FET is expected to facilitate single-stage repair by simplifying distal anastomosis.^5^ However, because TAR-FET does not excise the aneurysmal sac, questions persist regarding postoperative sac remodelling and the need for subsequent interventions. Although sac shrinkage after endovascular aortic repair (EVAR) for abdominal aortic aneurysms (AAA) is well-studied,^6^ similar data are lacking for true aortic arch aneurysms treated with TAR-FET. This study sought to evaluate the midterm incidence of sac shrinkage and additional interventions after TAR-FET and to identify anatomical predictors of sac remodelling using a novel sphericity index.

## METHODS

### Study population

Between July 2014 and March 2022, 31 patients underwent TAR-FET for true arch aneurysm at Hokkaido University Hospital. Two patients scheduled for staged surgery involving thoracic endovascular aortic repair (TEVAR) for extensive aortic arch aneurysms and 1 patient with substantial missing data were excluded. A total of 28 patients were included in this single-centre, retrospective, and observational study. Data on baseline characteristics (age, gender, and comorbidities), aneurysm morphology (maximum diameter, length, sphericity index, and saccular aneurysm), and surgical outcomes (procedure details, postoperative changes in aneurysm size, and additional interventions) were collected from medical records.

### Ethical statement

Informed consent was waived for 28 patients included in this study, as data were collected retrospectively from medical records and examination reports. The study protocol was approved by the Ethics Committee of Hokkaido University Hospital (approval number: 022-0242; date of approval: 16 November 2022).

No biological specimens or identifiable personal data were stored for repeated or indefinite future use. Therefore, the study does not involve the establishment of any biobank or database requiring ongoing ethical oversight, and compliance with the WMA Declaration of Taipei was not applicable.

### Follow-up

Postoperative computed tomography (CT) was performed in all patients prior to discharge, followed by additional scans at 6 months and 1 year. Subsequent evaluations were generally conducted on an annual basis.

### Aneurysm evaluation

**Figure 1.**
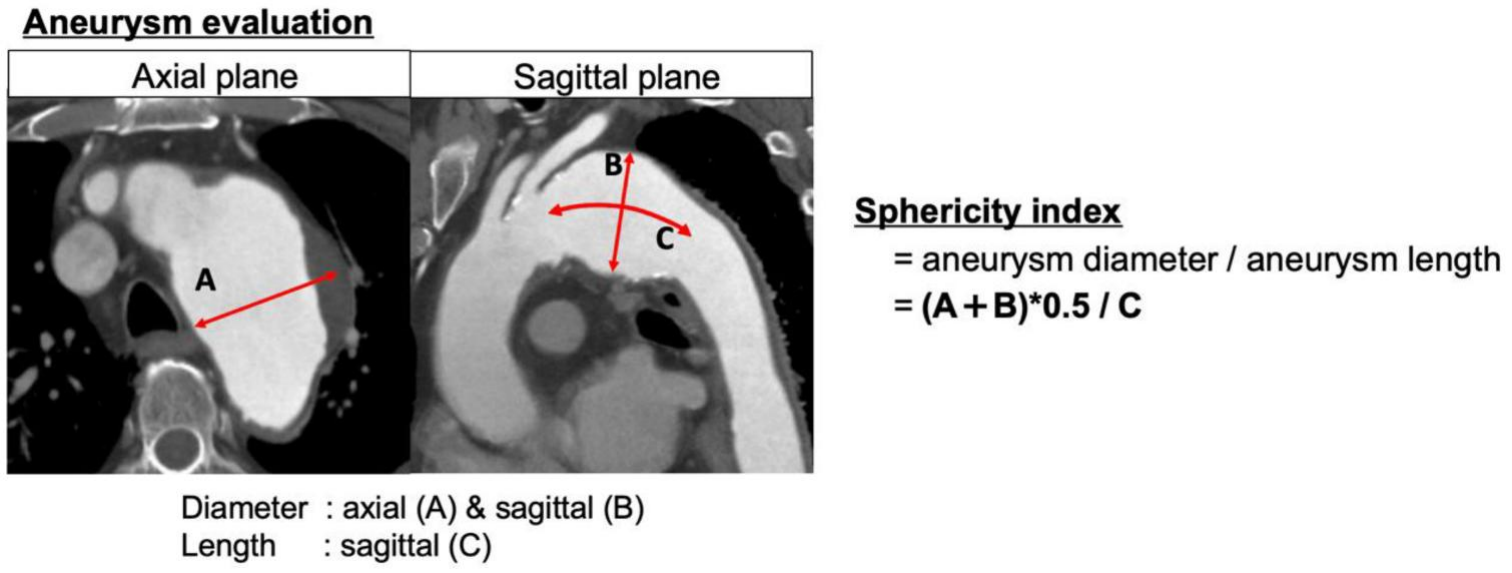
Schematic Illustration of Aneurysm Evaluation and Measurement

Previous studies evaluating sac shrinkage after EVAR for AAA have typically assessed changes in sac diameter using axial images alone.^6^ However, due to the complex 3-dimensional morphology of the aortic arch, axial CT image alone is insufficient to accurately assess sac size, particularly in the cranial and caudal directions. Therefore, in this study, aortic arch aneurysms were evaluated using both axial and sagittal planes, and aneurysm morphology was further qualified using a novel parameter, the sphericity index (**Figure 1**). The sphericity index was calculated by dividing the average of the maximum sac diameters measured on the axial and sagittal CT images by the sac length. Aneurysm length was measured on the sagittal plane along the aortic centreline, from the proximal inflection point—immediately preceding the dilated segment where the aortic diameter expanded to ≥40 mm—to the distal inflection point where it returned to a stable, normal caliber. Mildly ectatic segments without a clear transition to or from a dilated region were excluded. A higher sphericity index reflects a more protruding aneurysm. Aneurysms that expanded bilaterally in both the axial and sagittal planes were defined as fusiform, while those that protruded unilaterally in either plane were defined as saccular. Changes in sac diameter were categorized into 3 groups: shrinkage (a reduction of ≥5 mm in either the axial or sagittal plane), enlargement (an increase of ≥5 mm in either plane), or no change (a variation of <5 mm in both planes). The 5 mm threshold was adopted based on previous studies evaluating postoperative sac behaviour after EVAR for AAA, where a 5 mm change has been commonly used as a clinically meaningful threshold to define sac shrinkage or enlargement.^6^ The date on which a reduction of 5 mm or more was first observed on follow-up CT was defined as the date of sac shrinkage.

### Surgical procedures

The Frozenix J-Graft (Japan Lifeline Co. Ltd, Tokyo, Japan) was used as the FET prosthesis. The FET diameter was selected to be 110%-120% of the planned distal landing zone diameter, as measured on preoperative CT. The FET length was chosen to ensure that the distal end would not extend beyond the Th 8 vertebral level, in order to minimize the risk of spinal cord injury (SCI).^7^ If mobile plaques were present at the planned distal landing site, the FET was not used to avoid the risk of embolization. All procedures were performed through a median sternotomy. After establishing standard cardiopulmonary bypass with 2-stage right atrial drainage, circulatory arrest was induced by systemic cooling to a rectal temperature of 28 or 25°C. Following circulatory arrest, selective antegrade cerebral perfusion was initiated through cannulation of the supra-aortic vessels. The aortic arch was transected in zone 2, between the left common carotid artery and the left subclavian artery. The FET of the preselected size was inserted into the descending aorta and secured proximally with a continuous suture. A separate 4-branched graft was anastomosed proximally to the FET, and antegrade systemic perfusion was resumed. Supra-aortic vessel reconstruction was performed in the following order: left subclavian artery, left common carotid artery, and brachiocephalic artery. Finally, proximal anastomosis to the ascending aorta was completed.

### Statistical analysis

Continuous variables were reported as mean ± standard deviation (SD) or as median and interquartile range (IQR; first and third quartiles [Q1, Q3]), depending on data distribution. Normality was assessed using the Shapiro-Wilk test, which was deemed appropriate given the sample size. Categorical variables were presented as frequencies and percentages. The median follow-up duration was estimated using the inverse Kaplan-Meier method, in which patients who experienced an event were censored at the time of the event, while those without events were censored at the time of last follow-up. Competing risk analysis was performed to assess time-to-event outcomes, particularly for sac shrinkage, as additional treatments could significantly bias the effect of the initial intervention (TAR-FET). Based on the number of events and potential confounders,^8^ an age-adjusted Fine and Gray competing risk regression model was used to identify morphological predictors of sac shrinkage. Competing events were defined by the outcome: mortality and additional interventions for sac shrinkage, and mortality alone for additional interventions. To compare the effect sizes of predictors, hazard ratios per SD were also calculated for statistically significant variables.^9^ To evaluate predictive performance regarding sac shrinkage, time-dependent receiver operating characteristic (ROC) analysis was performed, including calculation of the area under the curve (AUC) and optimal threshold values. Participants lost to follow-up were censored at the time of last contact for survival analysis. All statistical analyses were performed using EZR (version 1. 54; Saitama Medical Center, Jichi Medical University, Saitama, Japan).^10^ A *P-*value < 0.05 was considered statistically significant.

## RESULTS

### Patient characteristics

**Table 1** shows the baseline characteristics of the patients. The median age was 74 years (IQR, 68-78), and 23 patients (82%) were male. No patients had connective tissue disorders such as Marfan syndrome. The median axial and sagittal diameters of the aneurysms were 57.0 mm (IQR, 49.0-60.3) and 52.9 mm (IQR, 44.4-57.5), respectively. The mean aneurysm length was 58.9 ± 20.7 mm. Saccular aneurysms accounted for 53.6% of all cases. The median sphericity index was 0.97.

**Table 1.** Patient Characteristics

Variables	Value (*n* = 28)
Age, years	74 (68-78)
Male, *n* (%)	23 (82)
Comorbidities, *n* (%)	
Hypertension	26 (92.9)
Hyperlipidaemia	16 (57.1)
Diabetes mellitus	7 (25)
Cerebrovascular disease	7 (25)
Coronary artery disease	4 (14.3)
Connective tissue disease	0 (0)
Haemodialysis	1 (3.5)
Oral antiplatelet or anticoagulant use, *n* (%)	14 (50)
Aneurysm morphology	
Axial diameter, mm	57 (49.0-60.3)
Sagittal diameter, mm	52.9 (44.4-57.5)
Mean diameter, mm	54.3 (47.2-58.6)
Aneurysm length, mm	58.9 ± 20.7
Saccular aneurysm, *n* (%)	15 (53.6)
Sphericity index	0.97 (0.74-1.23)

### Operative data

**Table 2** summarizes the operative data and the selected size of the FET. FETs with diameters ranging from 33 mm to 37 mm were evenly distributed, comprising 71% of all cases. Regarding length, 120 mm was the most frequently used, followed by 90 mm. The proximal end of the FET was anastomosed in zone 2 in most patients (79%), while the distal end was located at the level of T6 (46%) or T7 (39%).

**Table 2.** Operative Data

Variables	Value (*n* = 28)
Time, min	
Operation time	453 (371-512)
Cardiopulmonary bypass	235 (198-288)
Aortic cross-clamp	122 (106-150)
Selective cerebral perfusion	115 ± 35.7
Circulatory arrest	56.3 ± 14.3
Concomitant procedures, *n* (%)	
CABG	3 (10.7)
AVR	1 (3.6)
CABG＋AVR	1 (3.6)
CABG＋MAP	1 (3.6)
FET diameter, *n* (%)	
29 mm	3 (10.7)
31 mm	4 (14.3)
33 mm	7 (25.0)
35 mm	7 (25.0)
37 mm	6 (21.4)
39 mm	1 (3.6)
FET length, *n* (%)	
60 mm	1 (3.6)
90 mm	10 (35.7)
120 mm	14 (50.0)
150 mm	3 (10.7)
Proximal position of FET, *n* (%)	
Zone 1	5 (17.9)
Zone 2	22 (78.6)
Zone 3	1 (3.6)
Distal position of FET, *n* (%)	
T5	2 (7.1)
T6	13 (46.4)
T7	11 (39.3)
T8	2 (7.1)
Distal landing length, mm	41.7 ± 17.9

Abbreviations: AVR: aortic valve replacement; CABG: coronary artery bypass grafting; FET: frozen elephant trunk; MAP: mitral annuloplasty.

### Postoperative outcomes

**Figure 2.**
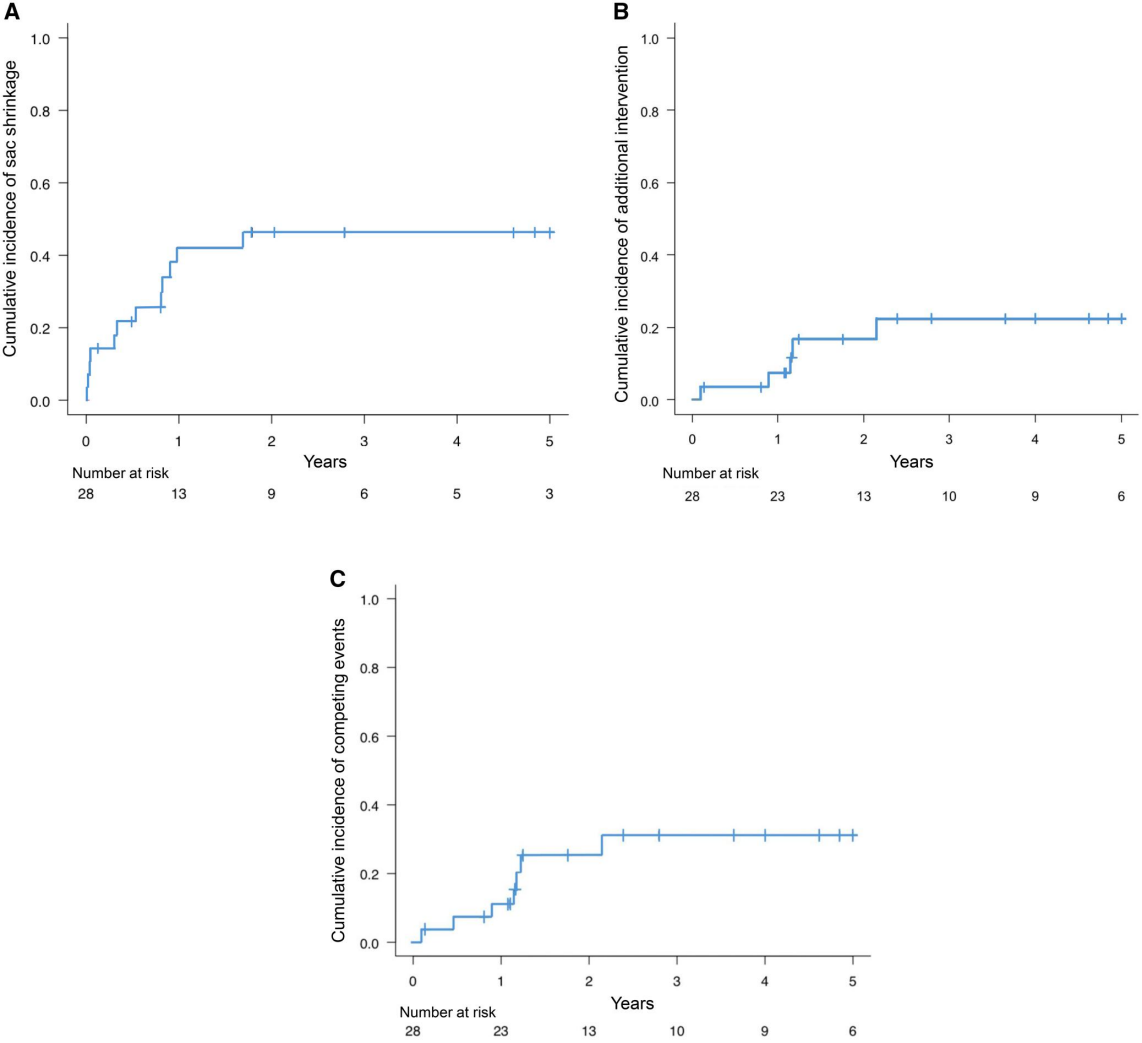
Composite figure consisting of three Kaplan–Meier curves: (A) Kaplan–Meier curve showing the cumulative incidence of sac shrinkage after TAR-FET over a 5-year period. The curve steadily increases, with approximately 50% of patients showing sac shrinkage by the end of follow-up. (B) Cumulative incidence of additional aortic reintervention following TAR-FET. The cumulative incidence of approximately 20% was observed. (C) Cumulative incidence of competing events (additional intervention and mortality) over time

**Table 3** illustrates the early postoperative outcomes. Cerebral infarction occurred in 2 patients (7%), and SCI was observed in 1 patient (3.5%). There were no cases of recurrent laryngeal nerve palsy or in-hospital mortality. The median follow-up duration was 3.6 years (95% confidence interval [CI], 1.24-5.15 years), with follow-up completion rates of 92%, 87%, and 84% at 1, 2, and 3 years, respectively. During this period, 2 patients died—one from cerebral haemorrhage and the other from AAA rupture. Sac shrinkage was observed in 12 cases (43%), with a median time to shrinkage of 5.3 months (IQR, 0.5-10.3 months). **Figure 2** shows the cumulative incidence of sac shrinkage , additional treatment , and competing events (mortality and additional treatment).The cumulative incidences of sac shrinkage at 1, 2, and 3 years were 42%, 47%, and 47%, respectively (**Figure 2A**). No patients experienced sac re-enlargement after initial shrinkage. In the comparison of preoperative morphology, the median aneurysm lengths were 42.0 mm (IQR, 37.2-58.4) in the shrinkage group and 69.1 mm (IQR, 53.7-74.9) in the no-shrinkage group. The corresponding sphericity indices were 1.27 (IQR, 0.95-1.55) and 0.79 (IQR, 0.72-1.06), respectively.

**Table 3.** Early Postoperative Outcomes

Variables	Value (*n* = 28)
Postoperative complications, *n* (%)	
Stroke	2 (7.1)
Spinal cord injury	1 (3.5)
Recurrent laryngeal nerve palsy	0 (0)
Deep sternum infection	1 (3.6)
Reexploration for bleeding	1 (3.6)
Newly initiated dialysis	2 (7.1)
Postoperative course	
ICU stay, days	2 (1-4)
Hospital stay, days	26 (17-38)

Abbreviation: ICU: intensive care unit.

Among the 16 patients without sac shrinkage (2 with enlargement and 14 with no change), 5 underwent additional TEVAR during follow-up (**Table S1**). Of these, 1 was treated for sac enlargement, 1 for distal stent graft-induced new entry (d-SINE), and 3 for prophylactic treatment of type Ib EL. Following these interventions, sac shrinkage was achieved in 2 patients. The cumulative incidences of additional intervention at 1, 2, and 3 years were 7%, 17%, and 22%, respectively (**Figure 2B**). The cumulative incidence of 7 competing events, including 2 deaths and 5 reinterventions, is shown in **Figure 2C**.

### Anatomical predictors of sac shrinkage

**Figure 3.**
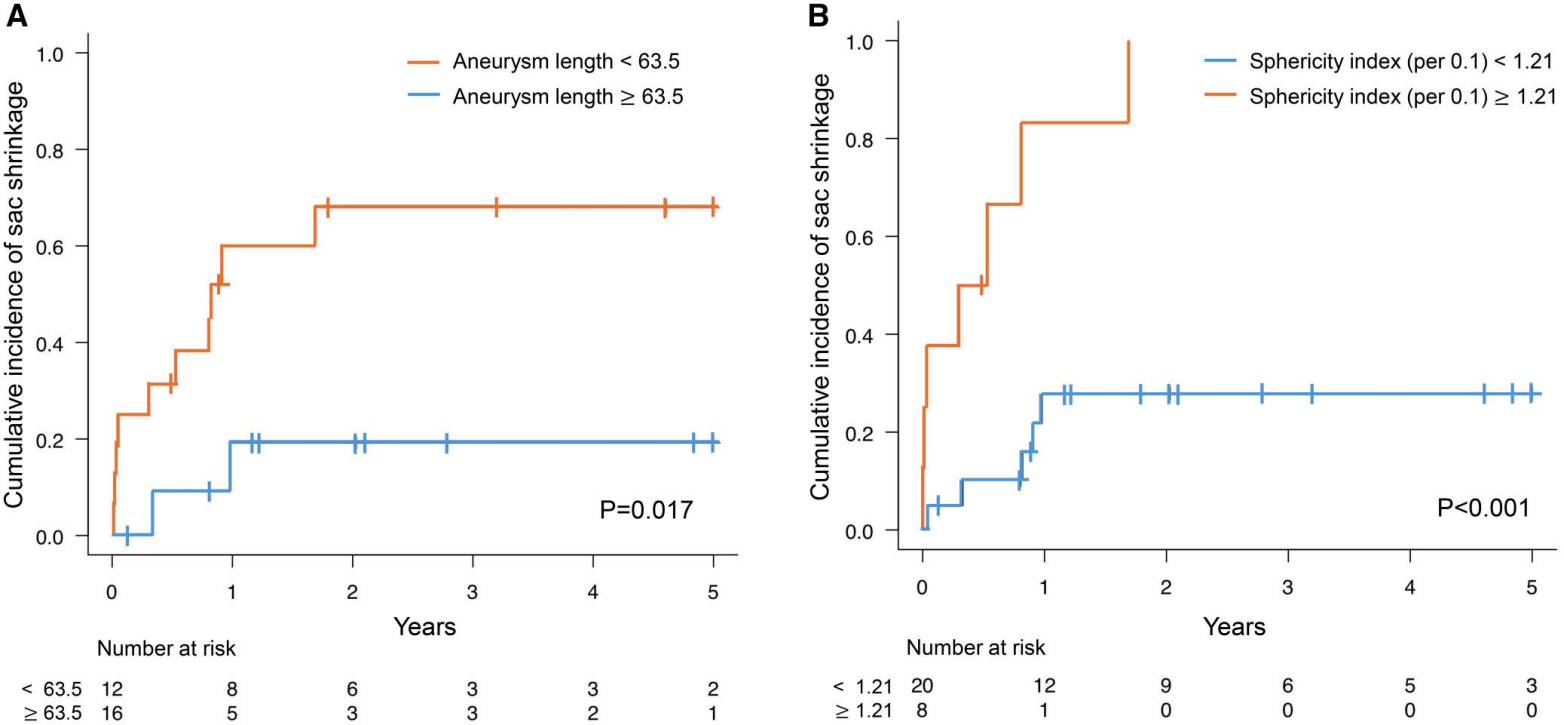
Composite figure consisting of two Kaplan–Meier curves: (A) Cumulative incidence of sac shrinkage stratified by aneurysm length (<63.5 mm vs. ≥63.5 mm), demonstrating significantly higher shrinkage rates in the shorter aneurysm group (p=0.017). (B) Cumulative incidence of sac shrinkage stratified by sphericity index (<1.21 vs. ≥1.21), showing significantly higher shrinkage in the more spherical aneurysms (p<0.001)

The results of the Fine and Gray analysis for sac shrinkage are presented in **Table 4**. Neither sac diameter nor distal landing position was significantly associated with sac shrinkage. In contrast, aneurysm length (age-adjusted subdistribution hazard ratio [SHR] = 0.96; 95% CI, 0.93-0.99; *p* = 0.018) and the sphericity index (per 0.1 increment; age-adjusted SHR = 1.38; 95% CI, 1.21-1.57; *p* < 0.001) were identified as independent predictor of postoperative sac shrinkage. The hazard ratios per SD were 2.33 for aneurysm length and 3.67 for the sphericity index (per 0.1 increment). **Figure 3** shows the results of time-dependent ROC analysis for sac shrinkage within 1 year after surgery. Aneurysm length had an AUC of 0.69, with a threshold value of 63.5, sensitivity of 0.82, and specificity of 0.59, while the sphericity index demonstrated a higher AUC of 0.79, with a threshold value of 1.21, sensitivity of 0.58, and specificity of 0.94 (**Figure 3**).

**Table 4.** Predictors of Sac Shrinkage

Factor	Age-adjusted SHR	95% confidence interval	*P*-value	SHR per SD
Axial diameter (per 1 mm)	1.06	0.98-1.15	0.15	
Sagittal diameter (per 1 mm)	1.01	0.92-1.11	0.80	
Mean diameter (per 1 mm)	1.03	0.94-1.13	0.47	
Saccular morphology	1.25	0.40-4.00	0.70	
Distal landing length (per 1 mm)	1.03	0.99-1.07	0.13	
Distal end position (T level)	0.55	0.25-1.20	0.13	
Aneurysm length (per 1 mm)	0.96	0.93-0.99	0.018	2.33
Sphericity index (per 0.1 increment)	1.38	1.21-1.57	< 0.001	3.67

Abbreviations: SD: standard deviation; SHR: subdistribution hazard ratio.

## DISCUSSIONS

Although TAR-FET for true aortic aneurysms has recently attracted attention, the rates of postoperative sac shrinkage and the need for additional interventions, as well as their predictors, remain important concerns for many surgeons. In this study, we evaluated the midterm results of TAR-FET for true aortic arch aneurysms. The 3-year cumulative incidences of sac shrinkage and additional interventions were 47% and 22%, respectively. Furthermore, a more protruding aneurysm—indicated by a higher sphericity index—was significantly associated with postoperative sac shrinkage, suggesting that such morphology may predict a favourable response to this procedure.

### Additional intervention after TAR-FET for true arch aneurysm

Tokunaga et al^11^ reported the results of TAR-FET for true arch aneurysms for 121 patients. Although changes in sac diameter were not addressed, 15 patients (12.4%) required additional interventions, including 13 TEVAR procedures for type Ib EL, inadequate distal landing, d-SINE, FET stenosis, and progression of the descending aortic aneurysm. The remaining 2 patients—one with a type II EL and the other with progressive descending aortic aneurysm—underwent coil embolization and thoraco-abdominal aortic replacement, respectively. In another study by Kandola et al,^12^ involving 36 cases of TAR-FET for true aneurysms, sac enlargement was observed in 3 cases (8%), and 5 patients (14%) required reintervention.

In the present study, 5 patients (18%) underwent additional intervention following TAR-FET, with a cumulative incidence of 22% at 3 years postoperatively. This reintervention rate is comparable to those reported in previous studies. Indications for additional TEVAR included sac enlargement in 2 cases, type Ib EL in 3, and d-SINE in 1 case. Notably, none of the patients who required additional TEVAR had experienced sac shrinkage. These findings suggest that the ability to predict sac shrinkage may help identify patients at lower risk for future reintervention.

### Sac shrinkage after TAR-FET for true arch aneurysm

Yamada et al^13^ reported that 18 of 25 patients (72%) achieved sac shrinkage after TAR-FET for aortic arch aneurysms; however, the definition of shrinkage was not clearly stated. Since accurate assessment of the aortic arch diameter is challenging using axial images alone, we defined sac shrinkage as a reduction of 5 mm or more in either the axial or sagittal dimension on CT imaging. Using this definition, sac shrinkage was observed in 12 cases (43%) in our cohort, with a 3-year cumulative shrinkage rate of 47%.

The predictors of sac shrinkage following TAR-FET for true arch aneurysm remain unclear. In EVAR for AAA, several morphological factors—including maximum aneurysm diameter, aneurysm length, and aneurysm configuration—have been identified as factors associated with sac shrinkage.^6^^,^^14^ In this study, we examined the relevance of these factors in the context of TAR-FET. Although no association was found between sac shrinkage and aneurysm diameter or saccular morphology, shorter aneurysm length was predictive of shrinkage. In addition, the sphericity index, an original parameter introduced in this study, showed a strong association with sac shrinkage. Its higher effective size and greater AUC compared to aneurysm length suggest that the sphericity index may serve as a more reliable predictor and may aid in clinical decision-making for patients undergoing TAR-FET for true arch aneurysm. Notably, the high specificity of 0.94 associated with the sphericity index may help avoid unnecessary interventions in patients unlikely to benefit from further procedures.

### Rationale for the prediction of sac shrinkage

The tendency for sac shrinkage in shorter aneurysms may be attributed to the lower number of branching vessels, such as the bronchial and intercostal arteries, as well as a higher likelihood of achieving an adequate distal landing zone. Adequate distal landing of the FET is considered to reduce the incidence of type Ib EL, thereby promoting sac shrinkage. However, in this study, the peripheral FET landing length was not found to be significantly associated with sac shrinkage.

Our findings suggest that more protruding aortic arch aneurysms are more likely to undergo sac shrinkage. Previous studies have reported that greater aneurysm protrusion may promote thrombus formation by altering intra-aneurysm flow velocity and shear stress.^15^ In addition, Dias et al^16^ demonstrated that intra-aneurysm thrombosis following EVAR contributes to sac shrinkage. In aortic arch aneurysms with a higher sphericity index, postoperative thrombosis may be more likely to occur, potentially contributing to sac shrinkage. Taken together, sac shrinkage after TAR-FET may be driven by a combination of favourable distal sealing in short aneurysms—owing to fewer branching vessels and a greater likelihood of adequate landing—and haemodynamic conditions in more protruding aneurysms that may promote mural thrombus formation. However, this study did not investigate the relationship between the sphericity index and mural thrombus formation, nor the changes in mural thrombus volume before and after the surgery. Further evaluation of these aspects will require additional case accumulation.

### Limitations

This study has several limitations. The small sample size and limited number of sac shrinkage events inevitably reduce statistical power and model stability. Nonetheless, the results appear relatively robust, supported by large effect sizes and favourable discriminative performance of the sphericity index. However, the stability and generalizability of the age-adjusted Fine and Gray competing risk model remain uncertain due to the limited number of events, and findings should be interpreted with caution.

We employed a competing risk framework to account for the impact of additional interventions on sac shrinkage. Without this, 2 patients who showed shrinkage only after secondary TEVAR might have been misclassified. While this approach was appropriate for our objective, the limited number of competing events may affect model reliability.

Due to the small sample, we included only age as a covariate to avoid overfitting. Although other clinical factors (eg, hypertension, smoking, connective tissue disease) could be potential confounders, their omission may weaken the model and should be considered in interpretation.

Finally, while our findings suggest a possible link between aneurysm morphology and thrombus formation, we did not assess thrombus volume or its changes. Future multicentre studies with larger cohorts and standardized imaging and clinical data are needed to validate these findings and improve predictive modelling after TAR-FET.

## CONCLUSIONS

At 3 years following TAR-FET for true aortic arch aneurysms, sac shrinkage was observed in 47% of patients, while 22% required additional intervention. Aneurysms that were shorter in length and more protruding in shape—reflected by a higher sphericity index—were more likely to undergo sac shrinkage. Although the observed associations were statistically significant, they should be interpreted as exploratory and hypothesis-generating, given the limited sample size and event count. Nonetheless, identifying preoperative morphological characteristics that may predict sac behaviour could help optimize patient selection and improve postoperative outcomes.

## Supplementary Material

ivaf200_Supplementary_Data

## Data Availability

All data generated or analyzed during this study are included in this published article.
