# Supplementary material for: Aneurysm Shape and Sac Shrinkage After Total Arch Replacement With Frozen Elephant Trunk for True Aortic Arch Aneurysm
Source: Interdiscip Cardiovasc Thorac Surg. 2025 Aug 21;40(9):ivaf200. doi: 10.1093/icvts/ivaf200 (PMC12448843; doi:10.1093/icvts/ivaf200)
Supplement: ivaf200_Supplementary_Data [file ivaf200_supplementary_data.zip › Supplementary Table 1.docx]

Supplementary Table. Characteristics of patients requiring additional TEVAR

| Case | Age | Sex | Aneurysm length, mm | Sphericity index | Sac change after TAR-FET | Days to additional TEVAR | Reason for additional TEVAR | Sac change after additional TEVAR |
| --- | --- | --- | --- | --- | --- | --- | --- | --- |
| 1 | 66 | Male | 71mm | 0.79 | No change | 35 | Type Ib EL | No change |
| 2 | 75 | Male | 90mm | 0.73 | No change | 427 | Type Ib EL | Shrinkage |
| 3 | 66 | Male | 54mm | 1.08 | No change | 325 | d-SINE | No change |
| 4 | 78 | Male | 80mm | 0.74 | No change | 418 | Type Ib EL | No change |
| 5 | 73 | Male | 51mm | 1.14 | Enlargement | 783 | Unexplained enlargement | Shrinkage |

d-SINE, distal stent-graft induced new entry; EL, endoleak; TEVAR, thoracic endovascular aortic repair ; TAR-FET, total arch replacement with frozen elephant trunk
